# Supplementary material for: Pyruvate Dehydrogenase Kinase 1 inhibition mediated oxidative phosphorylation enhancement in cartilage promotes osteoarthritis progression
Source: BMC Musculoskelet Disord. 2023 Jul 20;24:597. doi: 10.1186/s12891-023-06585-6 (PMC10357736; doi:10.1186/s12891-023-06585-6)
Supplement: Supplementary file 1 — Additional file 1. [file 12891_2023_6585_MOESM1_ESM.zip › KEGG.Gsea.1653622432464/KEGG_LYSOSOME.html]

Details for gene set KEGG\_LYSOSOME[GSEA]

|  || Dataset | 55457\_collapsed\_to\_symbols.cls.cls #OA\_versus\_normal.cls.cls #OA\_versus\_normal\_repos |
| Phenotype | cls.cls#OA\_versus\_normal\_repos |
| Upregulated in class | OA |
| GeneSet | KEGG\_LYSOSOME |
| Enrichment Score (ES) | 0.38321036 |
| Normalized Enrichment Score (NES) | 1.7891909 |
| Nominal p-value | 0.0 |
| FDR q-value | 0.012737292 |
| FWER p-Value | 0.149 |
Table: GSEA Results Summary

  

Fig 1: Enrichment plot: KEGG\_LYSOSOME      
 Profile of the Running ES Score & Positions of GeneSet Members on the Rank Ordered List

  

| SYMBOL | TITLE | RANK IN GENE LIST | RANK METRIC SCORE | RUNNING ES | CORE ENRICHMENT || 1 | CTSO | cathepsin O [Source:HGNC Symbol;Acc:HGNC:2542] | 63 | 0.970 | 0.0243 | Yes |
| 2 | ATP6V0D1 | ATPase H+ transporting V0 subunit d1 [Source:HGNC Symbol;Acc:HGNC:13724] | 135 | 0.872 | 0.0451 | Yes |
| 3 | CTSC | cathepsin C [Source:HGNC Symbol;Acc:HGNC:2528] | 227 | 0.801 | 0.0621 | Yes |
| 4 | CLN5 | CLN5 intracellular trafficking protein [Source:HGNC Symbol;Acc:HGNC:2076] | 248 | 0.785 | 0.0842 | Yes |
| 5 | GLB1 | galactosidase beta 1 [Source:HGNC Symbol;Acc:HGNC:4298] | 337 | 0.731 | 0.0993 | Yes |
| 6 | ATP6V0B | ATPase H+ transporting V0 subunit b [Source:HGNC Symbol;Acc:HGNC:861] | 382 | 0.713 | 0.1174 | Yes |
| 7 | GALC | galactosylceramidase [Source:HGNC Symbol;Acc:HGNC:4115] | 478 | 0.669 | 0.1301 | Yes |
| 8 | AGA | aspartylglucosaminidase [Source:HGNC Symbol;Acc:HGNC:318] | 647 | 0.606 | 0.1353 | Yes |
| 9 | ACP2 | "acid phosphatase 2, lysosomal [Source:HGNC Symbol;Acc:HGNC:123]" | 752 | 0.580 | 0.1446 | Yes |
| 10 | AP3S2 | adaptor related protein complex 3 subunit sigma 2 [Source:HGNC Symbol;Acc:HGNC:571] | 780 | 0.574 | 0.1598 | Yes |
| 11 | AP1S2 | adaptor related protein complex 1 subunit sigma 2 [Source:HGNC Symbol;Acc:HGNC:560] | 794 | 0.570 | 0.1760 | Yes |
| 12 | GUSB | glucuronidase beta [Source:HGNC Symbol;Acc:HGNC:4696] | 904 | 0.541 | 0.1838 | Yes |
| 13 | HYAL1 | hyaluronidase 1 [Source:HGNC Symbol;Acc:HGNC:5320] | 997 | 0.518 | 0.1922 | Yes |
| 14 | PPT1 | palmitoyl-protein thioesterase 1 [Source:HGNC Symbol;Acc:HGNC:9325] | 1042 | 0.508 | 0.2041 | Yes |
| 15 | AP4E1 | adaptor related protein complex 4 subunit epsilon 1 [Source:HGNC Symbol;Acc:HGNC:573] | 1065 | 0.502 | 0.2175 | Yes |
| 16 | DNASE2 | "deoxyribonuclease 2, lysosomal [Source:HGNC Symbol;Acc:HGNC:2960]" | 1133 | 0.489 | 0.2271 | Yes |
| 17 | GAA | alpha glucosidase [Source:HGNC Symbol;Acc:HGNC:4065] | 1149 | 0.486 | 0.2405 | Yes |
| 18 | SMPD1 | sphingomyelin phosphodiesterase 1 [Source:HGNC Symbol;Acc:HGNC:11120] | 1230 | 0.471 | 0.2485 | Yes |
| 19 | ATP6V1H | ATPase H+ transporting V1 subunit H [Source:HGNC Symbol;Acc:HGNC:18303] | 1304 | 0.457 | 0.2565 | Yes |
| 20 | HEXB | hexosaminidase subunit beta [Source:HGNC Symbol;Acc:HGNC:4879] | 1434 | 0.438 | 0.2597 | Yes |
| 21 | FUCA1 | alpha-L-fucosidase 1 [Source:HGNC Symbol;Acc:HGNC:4006] | 1506 | 0.428 | 0.2670 | Yes |
| 22 | NAGPA | N-acetylglucosamine-1-phosphodiester alpha-N-acetylglucosaminidase [Source:HGNC Symbol;Acc:HGNC:17378] | 1608 | 0.412 | 0.2715 | Yes |
| 23 | NAGA | alpha-N-acetylgalactosaminidase [Source:HGNC Symbol;Acc:HGNC:7631] | 1674 | 0.402 | 0.2786 | Yes |
| 24 | AP4M1 | adaptor related protein complex 4 subunit mu 1 [Source:HGNC Symbol;Acc:HGNC:574] | 1756 | 0.390 | 0.2840 | Yes |
| 25 | CTSH | cathepsin H [Source:HGNC Symbol;Acc:HGNC:2535] | 1803 | 0.384 | 0.2920 | Yes |
| 26 | PLA2G15 | phospholipase A2 group XV [Source:HGNC Symbol;Acc:HGNC:17163] | 1811 | 0.382 | 0.3030 | Yes |
| 27 | LIPA | "lipase A, lysosomal acid type [Source:HGNC Symbol;Acc:HGNC:6617]" | 1836 | 0.380 | 0.3126 | Yes |
| 28 | AP4S1 | adaptor related protein complex 4 subunit sigma 1 [Source:HGNC Symbol;Acc:HGNC:575] | 1841 | 0.380 | 0.3237 | Yes |
| 29 | ATP6AP1 | ATPase H+ transporting accessory protein 1 [Source:HGNC Symbol;Acc:HGNC:868] | 1866 | 0.377 | 0.3332 | Yes |
| 30 | AP3B1 | adaptor related protein complex 3 subunit beta 1 [Source:HGNC Symbol;Acc:HGNC:566] | 1883 | 0.375 | 0.3432 | Yes |
| 31 | CTSA | cathepsin A [Source:HGNC Symbol;Acc:HGNC:9251] | 2014 | 0.360 | 0.3439 | Yes |
| 32 | ASAH1 | N-acylsphingosine amidohydrolase 1 [Source:HGNC Symbol;Acc:HGNC:735] | 2201 | 0.338 | 0.3396 | Yes |
| 33 | CTSS | cathepsin S [Source:HGNC Symbol;Acc:HGNC:2545] | 2280 | 0.329 | 0.3434 | Yes |
| 34 | GM2A | GM2 ganglioside activator [Source:HGNC Symbol;Acc:HGNC:4367] | 2299 | 0.327 | 0.3518 | Yes |
| 35 | AP1B1 | adaptor related protein complex 1 subunit beta 1 [Source:HGNC Symbol;Acc:HGNC:554] | 2329 | 0.323 | 0.3593 | Yes |
| 36 | CTSG | cathepsin G [Source:HGNC Symbol;Acc:HGNC:2532] | 2457 | 0.307 | 0.3586 | Yes |
| 37 | GNPTAB | N-acetylglucosamine-1-phosphate transferase subunits alpha and beta [Source:HGNC Symbol;Acc:HGNC:29670] | 2648 | 0.285 | 0.3524 | Yes |
| 38 | SLC17A5 | solute carrier family 17 member 5 [Source:HGNC Symbol;Acc:HGNC:10933] | 2671 | 0.283 | 0.3592 | Yes |
| 39 | NAGLU | N-acetyl-alpha-glucosaminidase [Source:HGNC Symbol;Acc:HGNC:7632] | 2767 | 0.273 | 0.3600 | Yes |
| 40 | ARSB | arylsulfatase B [Source:HGNC Symbol;Acc:HGNC:714] | 2787 | 0.271 | 0.3667 | Yes |
| 41 | ATP6V0C | ATPase H+ transporting V0 subunit c [Source:HGNC Symbol;Acc:HGNC:855] | 2790 | 0.271 | 0.3747 | Yes |
| 42 | M6PR | "mannose-6-phosphate receptor, cation dependent [Source:HGNC Symbol;Acc:HGNC:6752]" | 2927 | 0.258 | 0.3718 | Yes |
| 43 | HEXA | hexosaminidase subunit alpha [Source:HGNC Symbol;Acc:HGNC:4878] | 3025 | 0.247 | 0.3717 | Yes |
| 44 | LAMP2 | lysosomal associated membrane protein 2 [Source:HGNC Symbol;Acc:HGNC:6501] | 3074 | 0.243 | 0.3753 | Yes |
| 45 | CLTB | clathrin light chain B [Source:HGNC Symbol;Acc:HGNC:2091] | 3131 | 0.237 | 0.3780 | Yes |
| 46 | GBA | glucosylceramidase beta [Source:HGNC Symbol;Acc:HGNC:4177] | 3327 | 0.219 | 0.3694 | Yes |
| 47 | ARSA | arylsulfatase A [Source:HGNC Symbol;Acc:HGNC:713] | 3334 | 0.218 | 0.3755 | Yes |
| 48 | CTNS | "cystinosin, lysosomal cystine transporter [Source:HGNC Symbol;Acc:HGNC:2518]" | 3337 | 0.218 | 0.3819 | Yes |
| 49 | AP3S1 | adaptor related protein complex 3 subunit sigma 1 [Source:HGNC Symbol;Acc:HGNC:2013] | 3479 | 0.204 | 0.3770 | Yes |
| 50 | SORT1 | sortilin 1 [Source:HGNC Symbol;Acc:HGNC:11186] | 3481 | 0.204 | 0.3831 | Yes |
| 51 | MAN2B1 | mannosidase alpha class 2B member 1 [Source:HGNC Symbol;Acc:HGNC:6826] | 3577 | 0.196 | 0.3816 | Yes |
| 52 | CLTCL1 | clathrin heavy chain like 1 [Source:HGNC Symbol;Acc:HGNC:2093] | 3631 | 0.191 | 0.3832 | Yes |
| 53 | TPP1 | tripeptidyl peptidase 1 [Source:HGNC Symbol;Acc:HGNC:2073] | 3735 | 0.182 | 0.3807 | No |
| 54 | SCARB2 | scavenger receptor class B member 2 [Source:HGNC Symbol;Acc:HGNC:1665] | 4048 | 0.152 | 0.3608 | No |
| 55 | GLA | galactosidase alpha [Source:HGNC Symbol;Acc:HGNC:4296] | 4172 | 0.140 | 0.3555 | No |
| 56 | AP3D1 | adaptor related protein complex 3 subunit delta 1 [Source:HGNC Symbol;Acc:HGNC:568] | 4198 | 0.138 | 0.3577 | No |
| 57 | CLTC | clathrin heavy chain [Source:HGNC Symbol;Acc:HGNC:2092] | 4330 | 0.126 | 0.3512 | No |
| 58 | ATP6V0A1 | ATPase H+ transporting V0 subunit a1 [Source:HGNC Symbol;Acc:HGNC:865] | 4371 | 0.123 | 0.3518 | No |
| 59 | ACP5 | "acid phosphatase 5, tartrate resistant [Source:HGNC Symbol;Acc:HGNC:124]" | 4456 | 0.117 | 0.3488 | No |
| 60 | NEU1 | neuraminidase 1 [Source:HGNC Symbol;Acc:HGNC:7758] | 4486 | 0.114 | 0.3499 | No |
| 61 | GGA2 | "golgi associated, gamma adaptin ear containing, ARF binding protein 2 [Source:HGNC Symbol;Acc:HGNC:16064]" | 4671 | 0.098 | 0.3385 | No |
| 62 | CLTA | clathrin light chain A [Source:HGNC Symbol;Acc:HGNC:2090] | 4718 | 0.094 | 0.3377 | No |
| 63 | CTSK | cathepsin K [Source:HGNC Symbol;Acc:HGNC:2536] | 4869 | 0.082 | 0.3285 | No |
| 64 | IDUA | alpha-L-iduronidase [Source:HGNC Symbol;Acc:HGNC:5391] | 5002 | 0.073 | 0.3204 | No |
| 65 | AP3M2 | adaptor related protein complex 3 subunit mu 2 [Source:HGNC Symbol;Acc:HGNC:570] | 5135 | 0.061 | 0.3119 | No |
| 66 | NPC2 | NPC intracellular cholesterol transporter 2 [Source:HGNC Symbol;Acc:HGNC:14537] | 5186 | 0.057 | 0.3097 | No |
| 67 | CTSD | cathepsin D [Source:HGNC Symbol;Acc:HGNC:2529] | 5243 | 0.053 | 0.3069 | No |
| 68 | GALNS | galactosamine (N-acetyl)-6-sulfatase [Source:HGNC Symbol;Acc:HGNC:4122] | 5302 | 0.048 | 0.3038 | No |
| 69 | ABCA2 | ATP binding cassette subfamily A member 2 [Source:HGNC Symbol;Acc:HGNC:32] | 5349 | 0.044 | 0.3016 | No |
| 70 | CTSF | cathepsin F [Source:HGNC Symbol;Acc:HGNC:2531] | 5373 | 0.042 | 0.3010 | No |
| 71 | LGMN | legumain [Source:HGNC Symbol;Acc:HGNC:9472] | 5433 | 0.038 | 0.2976 | No |
| 72 | NPC1 | NPC intracellular cholesterol transporter 1 [Source:HGNC Symbol;Acc:HGNC:7897] | 5480 | 0.034 | 0.2950 | No |
| 73 | AP1M2 | adaptor related protein complex 1 subunit mu 2 [Source:HGNC Symbol;Acc:HGNC:558] | 5573 | 0.026 | 0.2886 | No |
| 74 | SGSH | N-sulfoglucosamine sulfohydrolase [Source:HGNC Symbol;Acc:HGNC:10818] | 5668 | 0.018 | 0.2818 | No |
| 75 | HGSNAT | heparan-alpha-glucosaminide N-acetyltransferase [Source:HGNC Symbol;Acc:HGNC:26527] | 5809 | 0.008 | 0.2711 | No |
| 76 | ATP6V0A4 | ATPase H+ transporting V0 subunit a4 [Source:HGNC Symbol;Acc:HGNC:866] | 5968 | -0.002 | 0.2588 | No |
| 77 | CTSZ | cathepsin Z [Source:HGNC Symbol;Acc:HGNC:2547] | 6292 | -0.027 | 0.2344 | No |
| 78 | MANBA | mannosidase beta [Source:HGNC Symbol;Acc:HGNC:6831] | 6373 | -0.033 | 0.2291 | No |
| 79 | ABCB9 | ATP binding cassette subfamily B member 9 [Source:HGNC Symbol;Acc:HGNC:50] | 6442 | -0.038 | 0.2250 | No |
| 80 | SLC11A1 | solute carrier family 11 member 1 [Source:HGNC Symbol;Acc:HGNC:10907] | 7153 | -0.092 | 0.1722 | No |
| 81 | LAPTM5 | lysosomal protein transmembrane 5 [Source:HGNC Symbol;Acc:HGNC:29612] | 7168 | -0.093 | 0.1739 | No |
| 82 | TCIRG1 | "T cell immune regulator 1, ATPase H+ transporting V0 subunit a3 [Source:HGNC Symbol;Acc:HGNC:11647]" | 7600 | -0.127 | 0.1441 | No |
| 83 | CD164 | CD164 molecule [Source:HGNC Symbol;Acc:HGNC:1632] | 7757 | -0.138 | 0.1361 | No |
| 84 | IGF2R | insulin like growth factor 2 receptor [Source:HGNC Symbol;Acc:HGNC:5467] | 7829 | -0.143 | 0.1348 | No |
| 85 | DNASE2B | deoxyribonuclease 2 beta [Source:HGNC Symbol;Acc:HGNC:28875] | 7840 | -0.144 | 0.1384 | No |
| 86 | GGA3 | "golgi associated, gamma adaptin ear containing, ARF binding protein 3 [Source:HGNC Symbol;Acc:HGNC:17079]" | 8022 | -0.157 | 0.1289 | No |
| 87 | CLN3 | "CLN3 lysosomal/endosomal transmembrane protein, battenin [Source:HGNC Symbol;Acc:HGNC:2074]" | 8094 | -0.163 | 0.1283 | No |
| 88 | GGA1 | "golgi associated, gamma adaptin ear containing, ARF binding protein 1 [Source:HGNC Symbol;Acc:HGNC:17842]" | 8106 | -0.163 | 0.1324 | No |
| 89 | CTSV | cathepsin V [Source:HGNC Symbol;Acc:HGNC:2538] | 8359 | -0.183 | 0.1182 | No |
| 90 | LAMP3 | lysosomal associated membrane protein 3 [Source:HGNC Symbol;Acc:HGNC:14582] | 8400 | -0.185 | 0.1206 | No |
| 91 | CTSW | cathepsin W [Source:HGNC Symbol;Acc:HGNC:2546] | 8771 | -0.214 | 0.0982 | No |
| 92 | ATP6V0A2 | ATPase H+ transporting V0 subunit a2 [Source:HGNC Symbol;Acc:HGNC:18481] | 8843 | -0.221 | 0.0993 | No |
| 93 | SLC11A2 | solute carrier family 11 member 2 [Source:HGNC Symbol;Acc:HGNC:10908] | 9152 | -0.244 | 0.0826 | No |
| 94 | GNS | glucosamine (N-acetyl)-6-sulfatase [Source:HGNC Symbol;Acc:HGNC:4422] | 9345 | -0.259 | 0.0754 | No |
| 95 | AP1G1 | adaptor related protein complex 1 subunit gamma 1 [Source:HGNC Symbol;Acc:HGNC:555] | 9389 | -0.263 | 0.0799 | No |
| 96 | LAPTM4A | lysosomal protein transmembrane 4 alpha [Source:HGNC Symbol;Acc:HGNC:6924] | 9869 | -0.309 | 0.0518 | No |
| 97 | IDS | iduronate 2-sulfatase [Source:HGNC Symbol;Acc:HGNC:5389] | 10036 | -0.325 | 0.0486 | No |
| 98 | PPT2 | palmitoyl-protein thioesterase 2 [Source:HGNC Symbol;Acc:HGNC:9326] | 10103 | -0.331 | 0.0534 | No |
| 99 | CTSE | cathepsin E [Source:HGNC Symbol;Acc:HGNC:2530] | 10172 | -0.338 | 0.0583 | No |
| 100 | LAPTM4B | lysosomal protein transmembrane 4 beta [Source:HGNC Symbol;Acc:HGNC:13646] | 10218 | -0.343 | 0.0651 | No |
| 101 | CTSB | cathepsin B [Source:HGNC Symbol;Acc:HGNC:2527] | 10313 | -0.352 | 0.0684 | No |
| 102 | CTSL | cathepsin L [Source:HGNC Symbol;Acc:HGNC:2537] | 10817 | -0.407 | 0.0413 | No |
| 103 | CD63 | CD63 molecule [Source:HGNC Symbol;Acc:HGNC:1692] | 10850 | -0.411 | 0.0512 | No |
| 104 | LAMP1 | lysosomal associated membrane protein 1 [Source:HGNC Symbol;Acc:HGNC:6499] | 11142 | -0.449 | 0.0420 | No |
| 105 | AP1S1 | adaptor related protein complex 1 subunit sigma 1 [Source:HGNC Symbol;Acc:HGNC:559] | 12121 | -0.625 | -0.0156 | No |
| 106 | AP3B2 | adaptor related protein complex 3 subunit beta 2 [Source:HGNC Symbol;Acc:HGNC:567] | 12509 | -0.750 | -0.0233 | No |
| 107 | PSAP | prosaposin [Source:HGNC Symbol;Acc:HGNC:9498] | 12569 | -0.780 | -0.0043 | No |
| 108 | ENTPD4 | ectonucleoside triphosphate diphosphohydrolase 4 [Source:HGNC Symbol;Acc:HGNC:14573] | 12815 | -0.999 | 0.0066 | No |
Table: GSEA details [plain text format]

  

Fig 2: KEGG\_LYSOSOME      
 Blue-Pink O' Gram in the Space of the Analyzed GeneSet

  

Fig 3: KEGG\_LYSOSOME: Random ES distribution      
 Gene set null distribution of ES for **KEGG\_LYSOSOME**
